# Supplementary material for: Semantic code clone detection using hybrid intermediate representations and BiLSTM networks
Source: PLoS One. 2026 Jan 20;21(1):e0340971. doi: 10.1371/journal.pone.0340971 (PMC12818651; doi:10.1371/journal.pone.0340971)
Supplement: S1 File — List of Baf and Jimple features used in this work. (PDF) [file pone.0340971.s001.pdf]

Semantic Code Clone Detection Using Hybrid Intermediate Representations and BiLSTM Networks

M. Shahbaz Ismail[1], Sara Shahzad[1], Fahmi H. Quradaa[2*]

[1]Department of Computer Science, University of Peshawar, Peshawar, Pakistan

[2]Department of Computer Science, Aden Community College, Aden, Yemen

Emails: shahbazkhancs@uop.edu.pk, sara@uop.edu.pk,qurada@uop.edu.pk

1. List of Baf and Jimple Intermediate Representation Instructions

Table 1. BAF Instructions

ID	Instruction	Description
1	Load	Load variable from local variable
2	Store	Store variable into local variable
3	Inc	Increment local variable by constant
4	fieldget	Fetch field from object
5	fieldput	Set field in object
6	staticget	Get static field from class
7	staticput	Set static field in class
8	virtualinvoke	Invoke instance method; dispatch based on class
9	specialinvoke	Invoke instance method; direct invocation of instance initialization methods and methods of the current class and its supertypes
10	staticinvoke	Invoke a class (static) method
11	interfaceinvoke	Invoke interface method
12	Dup1	Duplicate the top operand stack value
13	Dup2	Duplicate the top one or two operand stack values
14	New	Create new object
15	Ifne	Jump if value1 <> value 2
16	Ifeq	Jump if value 1 = value 2
17	Ifge	Jump if value 1 >= value 2
18	Ifle	Jump if value 1 <= value 2
19	Ifgt	Jump if value 1 > value 2
20	Iflt	Jump if value 1 < value 2
21	Pop	Pop the top operand stack value
22	Push	Push variable into stack
23	Lookupswitch	Access jump table by key match and jump
24	Tableswitch	Access jump table by index and jump
25	Return	Return value from method
26	Ifcmpne	Branch if and only if Value1 <> value 2
27	Ifcmpeq	Branch if and only if Value1 = value 2
28	Ifcmpge	Branch if and only if value1 >= value2
29	Ifcmple	Branch if and only if Value1 <= value 2
30	Ifcmpgt	Branch if and only if value1 > value2
31	Ifcmplt	Branch if and only if value1 < value2
32	ifnull	Check if contains null
33	Cmpg	Compare value1 with value2 if value1 > value2 , value 1 pushed onto operand stack. otherwise, push 0 onto stack.
34	Cmp	Compare value1 with value2 if value1 > value2 , value 1 pushed onto operand stack. Otherwise if value1 < value2 then push -1 onto stack, otherwise, push 0 onto stack as value1 = value 2.
35	Cmpl	Compare value1 with value2 if value1 < value2 , value 1 pushed onto operand stack. otherwise, push 0 onto stack.
36	sub	Subtract two varaible

37	Add	Add two variables
38	div	Divide value1 by value2 that pops from stack
39	Mul	Multiply value1 with value2 that pops from stack
40	Rem	Remainder of division operation
41	Neg	Negate variable. If value is positive it will be converted into -value
42	Instanceof	instanceof determines whether objectref is an instance of T
43	Shl	Arithmetic shift left
44	Shr	Arithmetic shift right
45	Ushr	Logical shift right
46	Goto	Branch always
47	Throw	Throw exception or error
48	Checkcast	Check whether object is of given type
49	Newarray	Create new array
50	Newmultiarray	Create new multidimensional array
51	Arraylength	Get length of array
52	arraywrite	Write an array
53	arrayread	Read an array
54	Entermonitor	Enter monitor for object
55	Exitmonitor	Exit monitor for object
56	Nop	Do nothing
57	Breakpoint	Set a breakpoint at the instruction
58	And	Boolean AND
59	Or	Boolean OR
60	Xor	Boolean XOR

Table 2. Categorization of Jimple IR Statements

	Category	Feature Description
1	**Control Flow Graph**	Number of nodes in the CFG
2		Number of Edges in the CFG
3		Cyclomatic complexity of CFG = edges-nodes +2
4		Number of nodes have greater than 1 predecessors
5		Number of nodes have 1 predecessor
6		Number of nodes have greater than 1 successors
7		Number of nodes have 1 successor
8	**Block Control Flow Graph**	Number of blocks in Block CFG
9		Number of blocks have greater than 1 blocks predecessors
10		Number of blocks have 1 block predecessor
11		Number of blocks have greater than 1 blocks successors
12		Number of blocks have 1 block successor
13		Maximum Block (LOC)
14		Minimum Block (LOC)
15		Total length of the method (LOC)
16		Percentage of LOC in all Blocks
17	**Program Dependency Graph**	Number of PDG region
18		Number of strong regions in PDG
19		Number of weak regions in PDG
20		Number of region node in PDG
21		Number of PDGNODE node in PDG
22		Number of dependency Edges in PDG
23		Number of control flow edges in PDG
24		Number of dependency-back edges in PDG
25		Number of dependency edges between Region node and Region node in PDG
26		Number of dependency edges between Region node and Block node in PDG
27		Number of dependency edges between Block node and Region node in PDG
28		Number of dependency edges between Block node and Block node in PDG
29		Number of control flow edges between Region node and Region node in PDG
30		Number of control flow edges between Region node and Block node in PDG
31		Number of control flow edges between Block node and Region node in PDG

32		Number of control flow edges between Block node and Block node in PDG
33		Number of dependency-back edges between Region node and Region node in PDG
34		Number of dependency-back edges between Region node and Block node in PDG
35		Number of dependency-back edges between Block node and Region node in PDG
36		Number of dependency-back edges between Block node and Block node in PDG
