# Supplementary material for: Semantic code clone detection using hybrid intermediate representations and BiLSTM networks
Source: PLoS One. 2026 Jan 20;21(1):e0340971. doi: 10.1371/journal.pone.0340971 (PMC12818651; doi:10.1371/journal.pone.0340971)
Supplement: S2 File — Descriptive analysis of all clones types in BigCloneBench. (PDF) [file pone.0340971.s002.pdf]

Semantic Code Clone Detection Using Hybrid Intermediate Representations and BiLSTM Networks

M. Shahbaz Ismail[1], Sara Shahzad[1], Fahmi H. Quradaa[2*]

[1]Department of Computer Science, University of Peshawar, Peshawar, Pakistan

[2]Department of Computer Science, Aden Community College, Aden, Yemen

Emails: shahbazkhancs@uop.edu.pk, sara@uop.edu.pk,qurada@uop.edu.pk

1. Details of BigCloneBench dataset

Table 1. Details of BigCloneBench dataset

F_ID	# files	clones	T1	T2	T3	VST3	ST3	MT3	WT3	False_Postive
2	10317	386757	1551	9	385197	20	1407	2621	381149	36600
3	4341	827538	629	516	826393	515	2627	23025	800226	4065
4	21945	4329121	13770	3108	4312243	1193	4488	22431	4284131	184710
5	56	35			35			1	34	12
6	466	23435	4		23431	13	50	123	23245	3960
7	1028	13366	39	4	13323	21	197	1565	11540	13300
8	126	276	3	7	266	5		2	259	70
9	656	36			36				36	1209
10	1020	234954	152	64	234738	285	924	2257	231272	1738
11	62	209			209	1	3		205	
12	92	171	4	3	164	3	19	52	90	80
13	275	2775	7	5	2763	14	123	431	2195	576
14	2522	69006	110	7	68889	29	293	1816	66751	10856
15	71	325			325		4		321	15
17	174	210			210			2	208	441
18	106	736	5		731	5	4	42	680	102
19	348	4003	11		3992	2	5	73	3912	280
20	253	22155	11194	1	10960		1285	662	9013	
21	104	299	1		298	2		2	294	40
22	203	2628	1	1	2626	2	3	15	2606	98
23	326	69751	178	21	69552	90	219	878	68365	20
24	331	58247	37	20	58190	64	316	3896	53914	40
25	243	4357	4	3	4350	4	4	175	4167	158
26	127	231	1		230	0	15	43	172	213
27	514	77419	3537	8	73874	309	344	31	73190	480
28	220	19110	199	10	18901	64	112	177	18548	100
29	192	1378	15	2	1361	2	16	22	1321	141
30	1562	891772	195	32	891545	67	464	5176	885838	649
31	289	92573	1889	107	90577	96	81	735	89665	
32	291	27028	453	2	26573	3	389	199	25982	33
33	279	33148	58	6	33084	6	20	246	32812	22
34	475	104194	1483	25	102686	708	145	345	101488	70
35	785	320387	249	41	320097	347	53	502	319195	43
36	205	20892	90	34	20768	57	326	126	20259	6
37	365	11174	42	4	11128	17	56	218	10837	189
38	65	210			210	1		3	206	4
39	350	35245	253	27	34965	156	516	2436	31857	130
40	264	20100	482	2	19616	243	497	454	18422	49
41	443	86734	424	40	86270	223	1062	7713	77272	42
42	467	116403	46	19	116338	24	57	350	115907	3
43	324	10152	3	3	10146	22	160	361	9603	332

44	238	14028	10879		3149		149	10	2990	211
45	298	12561	1	3	12557	2	72	539	11944	119
Total	52818	7945129	47999	4134	7892996	4615	16505	79755	7792121	261206

2. Details of constructed dataset

Table 2. Details of constructed dataset

F_ID	# files	# clones	T1	T2	T3	VST3	ST3	MT3	WT3	F_Postive
2	9585	330076	1549	7	328520	20	1399	2395	324706	29904
3	3976	695607	625	509	694473	501	2491	21337	670144	3173
4	18954	3334632	11834	2977	3319821	1142	4018	18454	3296207	98460
5	53	35			35			1	34	8
6	439	18144	4		18140	11	34	81	18014	843
7	941	12880	39	4	12837	21	196	1526	11094	10808
8	125	231	3	7	221	5		1	215	0
9	570	28			28				28	702
10	910	174345	140	61	174144	275	856	1681	171332	1518
11	60	135			135		3		132	
23	299	40755	138	4	40613	73	85	353	40102	15
24	319	40722	36	19	40667	62	304	3490	36811	28
27	319	57289	3215	6	54068	309	342	13	53404	330
28	212	11935	149	10	11776	41	103	133	11499	92
30	1406	687373	161	30	687182	60	376	4414	682332	525
31	276	46338	70	104	46164	11	78	390	45685	
34	400	75077	1314	25	73738	672	113	228	72725	60
35	716	222101	219	41	221841	321	44	436	221040	0
37	266	10295	42	4	10249	16	55	181	9997	174
38	61	171			171	1	0	3	167	4
39	271	30876	247	27	30602	156	473	2211	27762	122
40	158	4095	71	1	4023	4	1	11	4007	12
41	396	53628	55	38	53535	95	665	4194	48581	36
42	433	102831	45	19	102767	24	57	324	102362	3
43	203	7021	3	3	7015	13	100	253	6649	278
44	236	66	1		65	0	1	5	59	186
45	272	11935	1	3	11931	2	72	539	11318	109
Total	**41856**	**5968621**	**19961**	**3899**	**5944761**	**3835**	**11866**	**62654**	**5866406**	**147390**
