# Supplementary material for: Semantic code clone detection using hybrid intermediate representations and BiLSTM networks
Source: PLoS One. 2026 Jan 20;21(1):e0340971. doi: 10.1371/journal.pone.0340971 (PMC12818651; doi:10.1371/journal.pone.0340971)
Supplement: Attachment — Submitted filename: Response to Reviewers.docx [file pone.0340971.s003.docx]

**Reviewer Response Report**

I would like to express my sincere gratitude to the Editor and the reviewer. Your valuable feedback and insights are greatly appreciated, and we are committed to addressing all your comments and suggestions to enhance the quality and impact of our work.

**Editor's Journal comments and responses**

- **Comment 1**: [*Please ensure that your manuscript meets PLOS ONE's style requirements, including those for file naming. The PLOS ONE style templates can be found at….*]

**Response** : We would like to inform you that we have adhered to PLOS ONE's style requirements as outlined in the PLOS ONE style templates.

- **Comment 2**: [*Please note that PLOS ONE has specific guidelines on code sharing for submissions in which author-generated code underpins the findings in the manuscript. In these cases, all author-generated code must be made available without restrictions upon publication of the work.***]**

**Response** : Thank you for your concerns regarding the  PLOS ONE guidelines on code sharing requirement. We assure you that we are fully committed to transparency and are willing to provide any details about the source code to reviewers upon request.

- **Comment 3**: [ *If the reviewer comments include a recommendation to cite specific previously published works, please review and evaluate these publications to determine whether they are relevant and should be cited. There is no requirement to cite these works unless the editor has indicated otherwise*.**]**

**Response** : We thank the reviewer for the suggestion. We reviewed the two recommended works, found them relevant, and have added them to the revised manuscript..

- **Comment** 4: [ *Please review your reference list to ensure that it is complete and correct. If you have cited papers that have been retracted, please include the rationale for doing so in the manuscript text, or remove these references and replace them with relevant current references. Any changes to the reference list should be mentioned in the rebuttal letter that accompanies your revised manuscript.***]**

**Response** : We have carefully reviewed the reference list to ensure that it is complete and accurate. Kindly refer to the revised version of the manuscript for these updates.

**Comments from Reviewer 1 and Responses**

- **Comment 1:** **[***The paper gives a clear and well organized contribution to the area of semantic code clone detection. The idea of using a mix of Baf and Jimple is creative and helps connect low level structure with higher level meaning in the code. The reasons for choosing these two representations are explained well, and the experiments support the choice. The model that uses a Siamese BiLSTM with attention adds more depth because it allows the system to focus on important parts of the code and understand context better. The evaluation is strong and covers different types of clones, and the comparisons with existing tools make the results more reliable..]*

**Response :** Thanks for your positive comments on our paper. Your comments and suggestions are responded to below

**Comment 2: [***The study works only with Java code, so the findings may not fully apply to other programming languages.* **]**

**Response :** We thank the reviewer for this valuable comment. We have clearly stated in the manuscript that our study focuses on the Java programming language and provided justification for this choice. We have also clarified in the Threats to Validity and Limitations section that our findings are primarily applicable to Java and may not directly generalize to other programming languages.

**Comment 3:** [ *The paper does not give much detail about how well the method scales when dealing with very large codebases or heavy workloads.]*

**Response :** We thank the reviewer for this insightful comment. The primary focus of this work is to propose and evaluate a novel code representation that combines two intermediate representations. A full scalability study was beyond the scope of this paper. We have clarified this in the revised manuscript and also highlighted scalability evaluation as an important direction for future work in the conclusion section.

**Comment 4:** [*Even though transformer models are mentioned for future research, the study does not compare its results with any transformer-based approaches, which leaves an important gap*.**]**

**Response :** We thank the reviewer for this valuable comment. In this work, our primary objective was to investigate the effectiveness of the proposed code representation and its integration with BiLSTM-based models. A direct comparison with transformer-based approaches was beyond the scope of this study due to differences in model architectures and computational requirements. We have clarified this limitation in the manuscript and strengthened the discussion by explicitly positioning transformer-based models as an important direction for future work.

**Comment 5:** [*The model still behaves like a black box, and the attention mechanism does not fully explain how decisions are being made*.**]**

**Response** : We thank the reviewer for this important comment. To address this concern, we have substantially revised the manuscript and added detailed explanations of the internal computations of the BiLSTM model, particularly on pages 14–17. These additions clarify how hidden states are computed and how attention weights are derived, thereby improving the transparency and interpretability of the model and reducing its black-box nature.
